# *rac*-4-{(*E*)-[1-Cyano-1-cyclo­hexyl-2-(1*H*-indol-3-yl)eth­yl]imino­meth­yl}benzonitrile

**DOI:** 10.1107/S1600536811049841

**Published:** 2011-11-25

**Authors:** Julien Letessier, Dieter Schollmeyer, Heiner Detert, Till Opatz

**Affiliations:** a University Mainz, Duesbergweg 10-14, 55128 Mainz, Germany

## Abstract

A phosphine-catalysed addition of gramine to an alkyl­idene­amino­nitrile gives the title compound, C_25_H_24_N_4_, in good yield. In the crystal, pairs of mol­ecules are connected *via* N—H⋯N hydrogen bonds into inversion dimers. The mol­ecules are characterized by a planar indole moiety [maximum deviation = 0.012 (1) Å], a chair conformation of the cyclo­hexane ring and an anti­periplanar conformation of the H atom on the cyclo­hexane and the adjacent cyano group.

## Related literature

For related structures, see: Son *et al.* (2008 ▶); Tacheva *et al.* (2010 ▶); Bergner *et al.* (2009 ▶); Patel *et al.* (2011 ▶)). For background to this work see: Dassonneville *et al.* (2011 ▶); Nissen & Detert (2011 ▶). For the synthesis, see: Somei *et al.* (1980 ▶). For synthetic applications of deprotonated aminonitriles, see: Opatz (2009 ▶); Meyer *et al.* (2005 ▶); in polysubstituted pyrroles, see: Bergner & Opatz (2007 ▶); in tetra­hydro­isoquinolines, see: Werner *et al.* (2007 ▶); Ferenc & Opatz (2008 ▶); Romek & Opatz (2010 ▶).
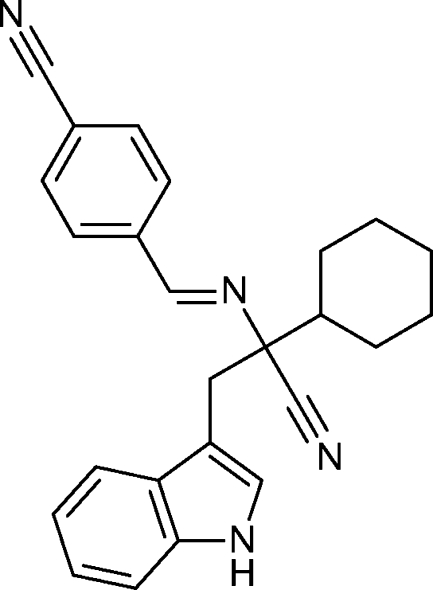


## Experimental

### 

#### Crystal data

C_25_H_24_N_4_
*M* *_r_* = 380.48
Triclinic, 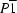

*a* = 9.0755 (6) Å
*b* = 10.4546 (8) Å
*c* = 11.8246 (8) Å
α = 92.958 (6)°
β = 105.995 (5)°
γ = 99.505 (6)°
*V* = 1058.09 (13) Å^3^
*Z* = 2
Mo *K*α radiation
μ = 0.07 mm^−1^
*T* = 193 K
0.34 × 0.17 × 0.13 mm

#### Data collection

Stoe IPDS 2T diffractometer
11005 measured reflections
5089 independent reflections
3183 reflections with *I* > 2σ(*I*)
*R*_int_ = 0.035

#### Refinement

*R*[*F* ^2^ > 2σ(*F* ^2^)] = 0.041
*wR*(*F* ^2^) = 0.107
*S* = 0.99
5089 reflections
262 parameters
H-atom parameters constrained
Δρ_max_ = 0.17 e Å^−3^
Δρ_min_ = −0.23 e Å^−3^

### 

Data collection: *X-AREA* (Stoe & Cie, 2001 ▶); cell refinement: *X-AREA*; data reduction: *X-RED* (Stoe & Cie, 2001 ▶); program(s) used to solve structure: *SIR97* (Altomare *et al.*, 1999 ▶); program(s) used to refine structure: *SHELXL97* (Sheldrick, 2008 ▶); molecular graphics: *PLATON* (Spek, 2009 ▶); software used to prepare material for publication: *PLATON*.

## Supplementary Material

Crystal structure: contains datablock(s) I, global. DOI: 10.1107/S1600536811049841/nc2256sup1.cif

Structure factors: contains datablock(s) I. DOI: 10.1107/S1600536811049841/nc2256Isup2.hkl

Supplementary material file. DOI: 10.1107/S1600536811049841/nc2256Isup3.cml

Additional supplementary materials:  crystallographic information; 3D view; checkCIF report

## Figures and Tables

**Table 1:** Hydrogen-bond geometry (Å, °)

*D*—H⋯*A*	*D*—H	H⋯*A*	*D*⋯*A*	*D*—H⋯*A*
N1—H1⋯N13^i^	0.88	2.17	2.9801 (18)	152

Symmetry code: (i) 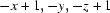.

**Fig. 1.**
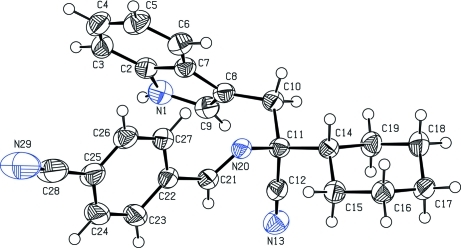
View of the title compound with labeling and displacement ellipsoids drawn at the 50% probability level.

**Fig. 2.**
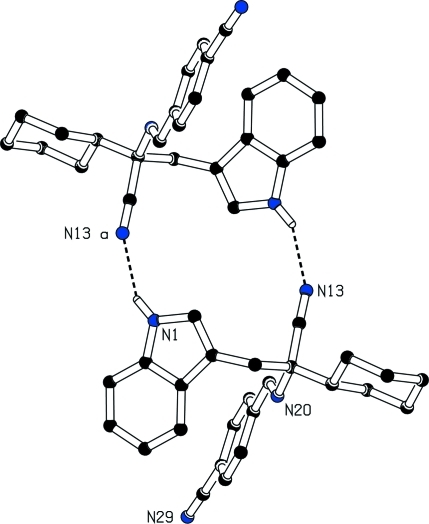
View of the centrosymmetric dimers with hydrogen bonding shown as dashed lines.

## supplementary crystallographic information

### Comment

The title compound was prepared as an intermediate in a new access to β-carbolines. A tributylphosphine catalyzed addition of gramine on alkylideneaminonitrile (Somei *et al.* (1980)) leads to the title compound (Fig. 1). The indole moiety is essentially planar (max. deviation at N1 0.012 (1) Å) and the cyclohexane ring adopts a chair conformation (Fig. 1). The hydrogen on C14 of the cyclohexane and the adjacent cyano group C12—N13 are in an anti-periplanar conformation (C12—C11—C14—H14: 178 °). Pairs of molecules form centrosymmetrical dimers (Fig. 2) connected *via* N1—H1···N13 hydrogen bridges (N1···N13 2.980 (2) Å) (Tab. 1).

### Experimental

4-[(*E*)-{[Cyano(cyclohexyl)methyl]imino}methyl]benzonitrile (90 mg, 1.0 equiv., 0.36 mmol) and gramine (66 mg, 1.05 equiv., 0.38 mmol) were dissolved in CH_3_CN (3 ml) in an argon-filled microwave reaction tube and P(Bu)_3_ (36 µ*L*, 0.40 equiv., 0.14 mmol) was added. The tube was sealed and irradiated (300 W) at 413 K for one hour (air cooling). The reaction mixture was diluted in CH_2_Cl_2_ (20 ml) and washed three times with sat aq. NaHCO_3_ (20 ml) and brine (10 ml). The organic layer was dried over MgSO_4_, filtered and the solvent was evaporated *in vacuo*. Purification by silica gel chromatography (petroleum ether / ethyl acetate / triethyl amine = 8 / 2 / 1) and recrystallization from petroleum ether / chloroform afforded the title compound as a pale yellow solid (104 mg, 76%).

### Refinement

Hydrogen atoms attached to carbons were placed at calculated positions with N—H = 0.88 Å, C—H = 0.95 Å (aromatic) or 0.98–0.99 Å (*sp*^3^ C-atom). All H atoms were refined in the riding-model approximation with isotropic displacement parameters (set at 1.2–1.5 times of the *U*_eq_ of the parent atom).

### Crystal data

**Table:**

C_25_H_24_N_4_	*Z* = 2
*M**_r_* = 380.48	*F*(000) = 404
Triclinic, *P*1	*D*_x_ = 1.194 Mg m^−^^3^
Hall symbol: -P 1	Melting point = 438–439 K
*a* = 9.0755 (6) Å	Mo *K*α radiation, λ = 0.71073 Å
*b* = 10.4546 (8) Å	Cell parameters from 7492 reflections
*c* = 11.8246 (8) Å	θ = 3.4–29.7°
α = 92.958 (6)°	µ = 0.07 mm^−^^1^
β = 105.995 (5)°	*T* = 193 K
γ = 99.505 (6)°	Block, colourless
*V* = 1058.09 (13) Å^3^	0.34 × 0.17 × 0.13 mm

### Data collection

**Table:**

Stoe IPDS 2T diffractometer	3183 reflections with *I* > 2σ(*I*)
Radiation source: sealed Tube	*R*_int_ = 0.035
graphite	θ_max_ = 28.0°, θ_min_ = 3.4°
Detector resolution: 6.67 pixels mm^-1^	*h* = −11→11
rotation method scans	*k* = −13→13
11005 measured reflections	*l* = −15→15
5089 independent reflections	

### Refinement

**Table:**

Refinement on *F*^2^	Primary atom site location: structure-invariant direct methods
Least-squares matrix: full	Secondary atom site location: difference Fourier map
*R*[*F*^2^ > 2σ(*F*^2^)] = 0.041	Hydrogen site location: inferred from neighbouring sites
*wR*(*F*^2^) = 0.107	H-atom parameters constrained
*S* = 0.99	*w* = 1/[σ^2^(*F*_o_^2^) + (0.052*P*)^2^] where *P* = (*F*_o_^2^ + 2*F*_c_^2^)/3
5089 reflections	(Δ/σ)_max_ < 0.001
262 parameters	Δρ_max_ = 0.17 e Å^−^^3^
0 restraints	Δρ_min_ = −0.23 e Å^−^^3^

### Special details

**Table:**

Experimental. IR (neat, ATR): ν (cm^-1^) = 3411 (br), 2927 (*s*), 2853 (*m*), 2227 (*m*), 1921 (w), 1643 (*m*), 1452 (*s*), 1095 (w), 1069 (w), 1009 (w), 892 (w), 834 (*s*), 771 (w),738 (*vs*).^1^H-NMR (400 MHz, CDCl_3_): δ = 8.05 (s, 1H, H1), 7.95 (s, 1H, H21), 7.65 (d, ^3^J_HH_ = 8.4 Hz, 2H, H23), 7.62 (d, ^3^J_HH_ = 8.4 Hz, 2H, H24), 7.44 (d, ^3^J_HH_ = 8.0 Hz, 1H, H6), 7.24 (m, 1H, H3), 7.09 (d, ^4^J_HH_ = 2. 4 Hz, 1H, H9), 7.04 (t, ^3^J_HH_ = 8.0 Hz, 1H, H5), 6.87 (t, ^3^J_HH_ = 7.5 Hz, 1H, H4), 3.49 (d, ^2^J_HH_ = 14.3 Hz, 1H, H10), 3.35 (d, ^2^J_HH_ = 14.3 Hz, 1H, 10-CH_2_), 2.26–1.66 (m, 6H, cyclohexane), 1.46–1.13 (m, 5H, cyclohexane).FD—MS: m/*z* (%) = 380.6 (100) [C_25_H_24_N_4_] ^+^, 381.6 (29).
Geometry. All e.s.d.'s (except the e.s.d. in the dihedral angle between two l.s. planes) are estimated using the full covariance matrix. The cell e.s.d.'s are taken into account individually in the estimation of e.s.d.'s in distances, angles and torsion angles; correlations between e.s.d.'s in cell parameters are only used when they are defined by crystal symmetry. An approximate (isotropic) treatment of cell e.s.d.'s is used for estimating e.s.d.'s involving l.s. planes.
Refinement. Refinement of *F*^2^ against ALL reflections. The weighted *R*-factor *wR* and goodness of fit *S* are based on *F*^2^, conventional *R*-factors *R* are based on *F*, with *F* set to zero for negative *F*^2^. The threshold expression of *F*^2^ > σ(*F*^2^) is used only for calculating *R*-factors(gt) *etc*. and is not relevant to the choice of reflections for refinement. *R*-factors based on *F*^2^ are statistically about twice as large as those based on *F*, and *R*- factors based on ALL data will be even larger.

### Fractional atomic coordinates and isotropic or equivalent isotropic displacement parameters (Å^2^)

**Table:**

	*x*	*y*	*z*	*U*_iso_*/*U*_eq_	
N1	0.58172 (14)	0.16501 (12)	0.34742 (10)	0.0446 (3)	
H1	0.6422	0.1093	0.3401	0.053*	
C2	0.51776 (15)	0.23949 (13)	0.26196 (11)	0.0388 (3)	
C3	0.52832 (18)	0.24696 (15)	0.14670 (12)	0.0486 (4)	
H3	0.5864	0.1944	0.1151	0.058*	
C4	0.45124 (19)	0.33346 (17)	0.08059 (12)	0.0545 (4)	
H4	0.4560	0.3403	0.0018	0.065*	
C5	0.36656 (18)	0.41101 (16)	0.12666 (13)	0.0523 (4)	
H5	0.3155	0.4703	0.0791	0.063*	
C6	0.35547 (16)	0.40333 (14)	0.24005 (12)	0.0434 (3)	
H6	0.2973	0.4567	0.2706	0.052*	
C7	0.43120 (15)	0.31584 (13)	0.30950 (11)	0.0356 (3)	
C8	0.44490 (15)	0.28356 (12)	0.42828 (11)	0.0359 (3)	
C9	0.53641 (16)	0.19104 (14)	0.44635 (12)	0.0419 (3)	
H9	0.5647	0.1505	0.5172	0.050*	
C10	0.37533 (15)	0.34143 (13)	0.51440 (11)	0.0391 (3)	
H10A	0.3701	0.4330	0.4990	0.047*	
H10B	0.4461	0.3428	0.5950	0.047*	
C11	0.20943 (15)	0.26983 (12)	0.51160 (10)	0.0339 (3)	
C12	0.22302 (15)	0.14187 (13)	0.55972 (11)	0.0369 (3)	
N13	0.23057 (15)	0.04430 (12)	0.59751 (10)	0.0487 (3)	
C14	0.13740 (15)	0.35027 (12)	0.58881 (10)	0.0346 (3)	
H14	0.1336	0.4365	0.5564	0.042*	
C15	−0.03045 (16)	0.28806 (14)	0.58034 (12)	0.0417 (3)	
H15A	−0.0947	0.2791	0.4968	0.050*	
H15B	−0.0323	0.1996	0.6067	0.050*	
C16	−0.10125 (17)	0.36810 (15)	0.65522 (12)	0.0450 (3)	
H16A	−0.1117	0.4526	0.6227	0.054*	
H16B	−0.2069	0.3213	0.6516	0.054*	
C17	−0.00124 (18)	0.39252 (14)	0.78303 (12)	0.0453 (3)	
H17A	0.0015	0.3086	0.8180	0.054*	
H17B	−0.0472	0.4484	0.8288	0.054*	
C18	0.16384 (18)	0.45897 (15)	0.79033 (12)	0.0488 (4)	
H18A	0.1614	0.5456	0.7606	0.059*	
H18B	0.2283	0.4723	0.8740	0.059*	
C19	0.23735 (17)	0.37809 (15)	0.71819 (11)	0.0446 (3)	
H19A	0.2488	0.2946	0.7525	0.054*	
H19B	0.3427	0.4255	0.7219	0.054*	
N20	0.11173 (12)	0.25599 (10)	0.38904 (8)	0.0344 (2)	
C21	0.06207 (15)	0.14545 (12)	0.33013 (10)	0.0365 (3)	
H21	0.0836	0.0694	0.3671	0.044*	
C22	−0.02917 (15)	0.13329 (13)	0.20417 (10)	0.0379 (3)	
C23	−0.1016 (2)	0.01174 (15)	0.14411 (12)	0.0529 (4)	
H23	−0.0948	−0.0640	0.1849	0.064*	
C24	−0.1835 (2)	−0.00046 (17)	0.02578 (13)	0.0644 (5)	
H24	−0.2321	−0.0841	−0.0149	0.077*	
C25	−0.19417 (19)	0.10979 (17)	−0.03294 (12)	0.0561 (4)	
C26	−0.12354 (17)	0.23217 (15)	0.02625 (12)	0.0478 (4)	
H26	−0.1322	0.3079	−0.0144	0.057*	
C27	−0.04120 (16)	0.24334 (13)	0.14366 (11)	0.0408 (3)	
H27	0.0080	0.3270	0.1839	0.049*	
C28	−0.2738 (3)	0.1007 (2)	−0.15794 (14)	0.0846 (7)	
N29	−0.3312 (3)	0.0976 (2)	−0.25669 (14)	0.1323 (11)	

### Atomic displacement parameters (Å^2^)

**Table:**

	*U*^11^	*U*^22^	*U*^33^	*U*^12^	*U*^13^	*U*^23^
N1	0.0387 (6)	0.0444 (7)	0.0524 (7)	0.0127 (5)	0.0132 (5)	0.0054 (5)
C2	0.0332 (7)	0.0404 (7)	0.0404 (7)	0.0009 (6)	0.0106 (5)	0.0017 (5)
C3	0.0439 (8)	0.0552 (9)	0.0458 (8)	−0.0023 (7)	0.0198 (6)	−0.0052 (6)
C4	0.0534 (9)	0.0698 (10)	0.0360 (7)	−0.0051 (8)	0.0149 (7)	0.0084 (7)
C5	0.0506 (9)	0.0617 (10)	0.0430 (7)	0.0064 (7)	0.0105 (7)	0.0189 (7)
C6	0.0403 (8)	0.0460 (8)	0.0446 (7)	0.0080 (6)	0.0123 (6)	0.0110 (6)
C7	0.0317 (7)	0.0382 (7)	0.0351 (6)	0.0016 (5)	0.0093 (5)	0.0049 (5)
C8	0.0332 (7)	0.0375 (7)	0.0352 (6)	0.0018 (5)	0.0091 (5)	0.0061 (5)
C9	0.0376 (7)	0.0443 (7)	0.0405 (7)	0.0043 (6)	0.0070 (6)	0.0087 (6)
C10	0.0378 (7)	0.0411 (7)	0.0349 (6)	−0.0009 (6)	0.0101 (5)	0.0015 (5)
C11	0.0352 (7)	0.0348 (6)	0.0295 (6)	0.0031 (5)	0.0069 (5)	0.0062 (5)
C12	0.0367 (7)	0.0414 (7)	0.0330 (6)	0.0072 (6)	0.0104 (5)	0.0056 (5)
N13	0.0535 (8)	0.0471 (7)	0.0502 (7)	0.0153 (6)	0.0177 (6)	0.0151 (6)
C14	0.0398 (7)	0.0337 (6)	0.0296 (6)	0.0046 (5)	0.0096 (5)	0.0061 (5)
C15	0.0402 (8)	0.0442 (7)	0.0401 (7)	0.0051 (6)	0.0127 (6)	0.0011 (6)
C16	0.0441 (8)	0.0479 (8)	0.0452 (7)	0.0085 (6)	0.0166 (6)	0.0047 (6)
C17	0.0606 (9)	0.0398 (7)	0.0425 (7)	0.0093 (7)	0.0255 (7)	0.0083 (6)
C18	0.0546 (9)	0.0541 (9)	0.0351 (7)	0.0038 (7)	0.0138 (6)	−0.0031 (6)
C19	0.0441 (8)	0.0545 (9)	0.0317 (6)	0.0059 (6)	0.0077 (6)	0.0012 (6)
N20	0.0360 (6)	0.0362 (6)	0.0297 (5)	0.0032 (4)	0.0090 (4)	0.0045 (4)
C21	0.0408 (7)	0.0348 (7)	0.0329 (6)	0.0039 (5)	0.0108 (5)	0.0047 (5)
C22	0.0393 (7)	0.0415 (7)	0.0314 (6)	0.0015 (6)	0.0116 (5)	0.0030 (5)
C23	0.0691 (10)	0.0420 (8)	0.0371 (7)	−0.0076 (7)	0.0086 (7)	0.0026 (6)
C24	0.0783 (12)	0.0567 (10)	0.0387 (8)	−0.0230 (9)	0.0068 (7)	−0.0012 (7)
C25	0.0538 (9)	0.0691 (10)	0.0324 (7)	−0.0151 (8)	0.0062 (6)	0.0066 (7)
C26	0.0488 (8)	0.0545 (9)	0.0369 (7)	0.0019 (7)	0.0103 (6)	0.0126 (6)
C27	0.0438 (8)	0.0410 (7)	0.0354 (6)	0.0025 (6)	0.0115 (6)	0.0029 (5)
C28	0.0929 (15)	0.0869 (14)	0.0417 (9)	−0.0396 (12)	−0.0022 (9)	0.0141 (8)
N29	0.163 (2)	0.1240 (16)	0.0438 (8)	−0.0756 (15)	−0.0218 (10)	0.0248 (9)

### Geometric parameters (Å, °)

**Table:**

N1—C2	1.3681 (17)	C15—H15A	0.9900
N1—C9	1.3715 (18)	C15—H15B	0.9900
N1—H1	0.8800	C16—C17	1.517 (2)
C2—C3	1.3976 (18)	C16—H16A	0.9900
C2—C7	1.4051 (19)	C16—H16B	0.9900
C3—C4	1.378 (2)	C17—C18	1.521 (2)
C3—H3	0.9500	C17—H17A	0.9900
C4—C5	1.391 (2)	C17—H17B	0.9900
C4—H4	0.9500	C18—C19	1.521 (2)
C5—C6	1.3773 (19)	C18—H18A	0.9900
C5—H5	0.9500	C18—H18B	0.9900
C6—C7	1.3992 (19)	C19—H19A	0.9900
C6—H6	0.9500	C19—H19B	0.9900
C7—C8	1.4382 (16)	N20—C21	1.2633 (16)
C8—C9	1.364 (2)	C21—C22	1.4786 (17)
C8—C10	1.4895 (19)	C21—H21	0.9500
C9—H9	0.9500	C22—C23	1.3875 (19)
C10—C11	1.5576 (17)	C22—C27	1.3897 (18)
C10—H10A	0.9900	C23—C24	1.380 (2)
C10—H10B	0.9900	C23—H23	0.9500
C11—N20	1.4612 (15)	C24—C25	1.379 (2)
C11—C12	1.4910 (17)	C24—H24	0.9500
C11—C14	1.5489 (18)	C25—C26	1.388 (2)
C12—N13	1.1394 (16)	C25—C28	1.446 (2)
C14—C15	1.5282 (18)	C26—C27	1.3719 (18)
C14—C19	1.5330 (17)	C26—H26	0.9500
C14—H14	1.0000	C27—H27	0.9500
C15—C16	1.520 (2)	C28—N29	1.136 (2)
			
C2—N1—C9	109.06 (12)	C14—C15—H15B	109.2
C2—N1—H1	125.5	H15A—C15—H15B	107.9
C9—N1—H1	125.5	C17—C16—C15	111.34 (12)
N1—C2—C3	130.63 (14)	C17—C16—H16A	109.4
N1—C2—C7	107.47 (11)	C15—C16—H16A	109.4
C3—C2—C7	121.90 (13)	C17—C16—H16B	109.4
C4—C3—C2	117.47 (15)	C15—C16—H16B	109.4
C4—C3—H3	121.3	H16A—C16—H16B	108.0
C2—C3—H3	121.3	C16—C17—C18	110.28 (11)
C3—C4—C5	121.45 (13)	C16—C17—H17A	109.6
C3—C4—H4	119.3	C18—C17—H17A	109.6
C5—C4—H4	119.3	C16—C17—H17B	109.6
C6—C5—C4	121.14 (14)	C18—C17—H17B	109.6
C6—C5—H5	119.4	H17A—C17—H17B	108.1
C4—C5—H5	119.4	C19—C18—C17	111.34 (12)
C5—C6—C7	119.02 (15)	C19—C18—H18A	109.4
C5—C6—H6	120.5	C17—C18—H18A	109.4
C7—C6—H6	120.5	C19—C18—H18B	109.4
C6—C7—C2	119.01 (12)	C17—C18—H18B	109.4
C6—C7—C8	133.73 (13)	H18A—C18—H18B	108.0
C2—C7—C8	107.25 (11)	C18—C19—C14	111.10 (12)
C9—C8—C7	106.01 (12)	C18—C19—H19A	109.4
C9—C8—C10	127.18 (11)	C14—C19—H19A	109.4
C7—C8—C10	126.80 (12)	C18—C19—H19B	109.4
C8—C9—N1	110.21 (12)	C14—C19—H19B	109.4
C8—C9—H9	124.9	H19A—C19—H19B	108.0
N1—C9—H9	124.9	C21—N20—C11	121.14 (11)
C8—C10—C11	115.35 (10)	N20—C21—C22	120.67 (12)
C8—C10—H10A	108.4	N20—C21—H21	119.7
C11—C10—H10A	108.4	C22—C21—H21	119.7
C8—C10—H10B	108.4	C23—C22—C27	119.03 (12)
C11—C10—H10B	108.4	C23—C22—C21	120.53 (12)
H10A—C10—H10B	107.5	C27—C22—C21	120.42 (12)
N20—C11—C12	112.70 (10)	C24—C23—C22	120.77 (14)
N20—C11—C14	108.71 (10)	C24—C23—H23	119.6
C12—C11—C14	108.63 (9)	C22—C23—H23	119.6
N20—C11—C10	107.39 (9)	C25—C24—C23	119.43 (14)
C12—C11—C10	108.49 (11)	C25—C24—H24	120.3
C14—C11—C10	110.94 (10)	C23—C24—H24	120.3
N13—C12—C11	178.41 (14)	C24—C25—C26	120.46 (13)
C15—C14—C19	110.85 (10)	C24—C25—C28	121.09 (15)
C15—C14—C11	112.32 (10)	C26—C25—C28	118.41 (15)
C19—C14—C11	112.82 (11)	C27—C26—C25	119.74 (13)
C15—C14—H14	106.8	C27—C26—H26	120.1
C19—C14—H14	106.8	C25—C26—H26	120.1
C11—C14—H14	106.8	C26—C27—C22	120.56 (13)
C16—C15—C14	112.13 (11)	C26—C27—H27	119.7
C16—C15—H15A	109.2	C22—C27—H27	119.7
C14—C15—H15A	109.2	N29—C28—C25	176.98 (19)
C16—C15—H15B	109.2		
			
C9—N1—C2—C3	−179.37 (14)	C10—C11—C14—C15	175.47 (10)
C9—N1—C2—C7	0.76 (14)	N20—C11—C14—C19	−176.23 (10)
N1—C2—C3—C4	−179.33 (13)	C12—C11—C14—C19	60.80 (13)
C7—C2—C3—C4	0.5 (2)	C10—C11—C14—C19	−58.36 (13)
C2—C3—C4—C5	0.3 (2)	C19—C14—C15—C16	53.39 (15)
C3—C4—C5—C6	−0.6 (2)	C11—C14—C15—C16	−179.38 (10)
C4—C5—C6—C7	0.1 (2)	C14—C15—C16—C17	−55.08 (15)
C5—C6—C7—C2	0.72 (19)	C15—C16—C17—C18	56.49 (16)
C5—C6—C7—C8	179.62 (13)	C16—C17—C18—C19	−57.64 (16)
N1—C2—C7—C6	178.85 (11)	C17—C18—C19—C14	56.67 (15)
C3—C2—C7—C6	−1.03 (19)	C15—C14—C19—C18	−53.95 (15)
N1—C2—C7—C8	−0.32 (14)	C11—C14—C19—C18	179.09 (11)
C3—C2—C7—C8	179.79 (12)	C12—C11—N20—C21	−7.59 (17)
C6—C7—C8—C9	−179.23 (14)	C14—C11—N20—C21	−128.07 (12)
C2—C7—C8—C9	−0.23 (14)	C10—C11—N20—C21	111.83 (13)
C6—C7—C8—C10	−0.2 (2)	C11—N20—C21—C22	−176.49 (11)
C2—C7—C8—C10	178.80 (12)	N20—C21—C22—C23	−171.10 (14)
C7—C8—C9—N1	0.71 (14)	N20—C21—C22—C27	10.1 (2)
C10—C8—C9—N1	−178.32 (12)	C27—C22—C23—C24	0.6 (2)
C2—N1—C9—C8	−0.93 (15)	C21—C22—C23—C24	−178.30 (15)
C9—C8—C10—C11	−90.70 (16)	C22—C23—C24—C25	−0.5 (3)
C7—C8—C10—C11	90.47 (15)	C23—C24—C25—C26	−0.1 (3)
C8—C10—C11—N20	−51.87 (14)	C23—C24—C25—C28	177.63 (18)
C8—C10—C11—C12	70.21 (13)	C24—C25—C26—C27	0.7 (3)
C8—C10—C11—C14	−170.53 (10)	C28—C25—C26—C27	−177.12 (16)
N20—C11—C12—N13	−93 (5)	C25—C26—C27—C22	−0.6 (2)
C14—C11—C12—N13	27 (5)	C23—C22—C27—C26	0.0 (2)
C10—C11—C12—N13	148 (5)	C21—C22—C27—C26	178.88 (13)
N20—C11—C14—C15	57.61 (12)	C24—C25—C28—N29	−137 (6)
C12—C11—C14—C15	−65.36 (13)	C26—C25—C28—N29	41 (6)

### Hydrogen-bond geometry (Å, °)

**Table:**

*D*—H···*A*	*D*—H	H···*A*	*D*···*A*	*D*—H···*A*
N1—H1···N13^i^	0.88	2.17	2.9801 (18)	152

Symmetry codes: (i) −*x*+1, −*y*, −*z*+1.
